# Supplementary material for: Single Cell Transcriptomic and Chromatin Profiles Suggest Layer Vb Is the Only Layer With Shared Excitatory Cell Types in the Medial and Lateral Entorhinal Cortex
Source: Front Neural Circuits. 2022 Jan 26;15:806154. doi: 10.3389/fncir.2021.806154 (PMC8826650; doi:10.3389/fncir.2021.806154)
Supplement: Supplementary file 14 [file Data_Sheet_1.ZIP › Inh_vs_Exc_Motifs/homerResults/motif17.similar.html]

motif17

## Information for motif17

G
A
T
C
A
C
T
G
A
T
G
C
A
C
G
T
A
C
T
G
A
G
C
T
G
A
C
T
A
G
T
C
T
C
A
G
A
T
C
G
  
Reverse Opposite:  

A
T
G
C
A
G
T
C
T
C
A
G
C
T
G
A
T
C
G
A
G
T
A
C
C
G
T
A
T
A
C
G
A
G
T
C
C
T
A
G
  

|  |  |
| --- | --- |
| p-value: | 1e-15 |
| log p-value: | -3.540e+01 |
| Information Content per bp: | 1.822 |
| Number of Target Sequences with motif | 86.0 |
| Percentage of Target Sequences with motif | 0.70% |
| Number of Background Sequences with motif | 98.2 |
| Percentage of Background Sequences with motif | 0.25% |
| Average Position of motif in Targets | 301.7 +/- 270.0bp |
| Average Position of motif in Background | 392.1 +/- 369.1bp |
| Strand Bias (log2 ratio + to - strand density) | 0.4 |
| Multiplicity (# of sites on avg that occur together) | 1.00 |
| Motif File: | file (matrix) reverse opposite |

### Similar de novo motifs found

|  |  |  |  |  |  |  |  |
| --- | --- | --- | --- | --- | --- | --- | --- |
| Rank | Match Score | Redundant Motif | P-value | log P-value | % of Targets | % of Background | Motif file |
| 1 | 0.612 | C G T A A T G C C G T A A C T G A G T C A C T G A C T G G A T C | 1e-10 | -23.745428 | 5.20% | 4.00% | motif file (matrix) |
